# Incorporation of CNF with Different Charge Property into PVP Hydrogel and Its Characteristics

**DOI:** 10.3390/nano11020426

**Published:** 2021-02-08

**Authors:** Wanhee Im, Shin Young Park, Sooim Goo, Simyub Yook, Hak Lae Lee, Guihua Yang, Hye Jung Youn

**Affiliations:** 1 Research Institute of Agriculture and Life Sciences, Seoul National University, 1 Gwanak-ro, Gwanak-gu, Seoul 08826, Korea; iwh1009@moorim.co.kr (W.I.); lhakl@snu.ac.kr (H.L.L.); 2 R&D Institute, Moorim P&P Co., 3-36 Ubonggangyang-ro, Onsan-eup, Ulju-gun, Ulsan 45011, Korea; 3 Department of Forest Sciences, Seoul National University, 1 Gwanak-ro, Gwanak-gu, Seoul 08826, Korea; sypk1992@snu.ac.kr; 4 Department of Agriculture, Forestry and Bioresources, Seoul National University, 1 Gwanak-ro, Gwanak-gu, Seoul 08826, Korea; ksi960@snu.ac.kr (S.G.); breedingcrops@snu.ac.kr (S.Y.); 5 State Key Laboratory of Biobased Material and Green Papermaking (Shandong Academy of Sciences), Qilu University of Technology, 3501 Daxue Rd, Changqing District, Jinan 250353, China; ygh@qlu.edu.cn

**Keywords:** cellulose nanofibrils, chemical pretreatment, electrostatic property, hydrogel, reinforcement, swelling ability

## Abstract

Cellulose nanofibril (CNF)-added polyvinylpyrrolidone (PVP) hydrogels were prepared using different types of CNFs and their properties were investigated. CNFs with different morphology and surface charge properties were prepared through quaternization and carboxymethylation pretreatments. The quaternized CNF exhibited the narrow and uniform width, and higher viscoelastic property compared to untreated and carboxymethylated CNF. When CNF was incorporated to PVP hydrogel, gel contents of all hydrogels were similar, irrespective of CNF addition quantity or CNF type. However, the absorptivity of the hydrogels in a swelling medium increased by adding CNF. In particular, the quaternized CNF-added PVP hydrogel exhibited the highest swelling ability. Unlike that of hydrogels with untreated and carboxymethylated CNFs, the storage modulus of PVP hydrogels after swelling significantly increased with an increase in the content of the quaternized CNF. These indicate that a PVP hydrogel with a high absorptivity and storage modulus can be prepared by incorporating the proper type of CNF.

## 1. Introduction

Since the “nano” concept has received great attention in many scientific studies, interests in cellulose nanofibrils (CNF) have been increasing so as to use the full potential of cellulose. There have been considerable scientific and commercial interests in the use of CNF in a wide range of applications, such as paper strength additive, transparent film, reinforcing composites, packaging materials, and so on [1,2,3,4]. These wide interests are thanks to its biodegradability, high strength, and lightweight properties of CNF [5,6]. In order to decrease high-energy consumption to produce CNF, various chemical pretreatments including carboxymethylation, TEMPO-oxidation, and quaternization have been studied and reviewed [7,8,9]. Chemical pretreatments are all aimed at substituting the hydroxyl groups with anionic or cationic charge groups, and these charge groups lead to electrostatic repulsion and fiber swelling, which weaken the hydrogen bonds between the fibers and facilitate nano-fibrillation of pulp fiber. Introduced charged groups impart new properties that are different from the untreated cellulose material. Onyianta et al. [10] compared the effect of CNF prepared by different pretreatment methods on the formation of crosslinked hydrogel. This study shows that the network structure, storage modulus and swelling degree of the CNF composite hydrogel are influenced by the presence or absence of anionic surface charge on CNF. In addition, Fukuzumi et al. [11] prepared three CNFs with different average length by controlling anionic group contents. It was shown that the CNF length influenced optical, mechanical, and gas barrier properties of CNF self-standing films. This indicated that the end-use properties of CNF products can be affected by surface charge and structural properties of CNF.

Among the diverse application fields of CNF-based products, CNF hydrogels have great potential for application in such fields as tissue engineering, drug delivery, sorbents, sensors, and purification [12]. The CNF hydrogels are commonly produced by adding salt or lowering the pH of a CNF suspension. Because of counter ion-driven charge screening of the charged groups of fibrils, CNF suspension readily forms gels via reduction of surface charge. However, low mechanical strength and unstable dimensions of pure CNF hydrogels that associated with the changes in water content severely restrict their range of application [13]. Therefore, CNF have been explored as reinforcing materials of synthetic polymer hydrogels to improve the mechanical properties and introduce functionality [14]. For example, Liu et al. [15] investigated the use of CNF prepared by a sulfuric acid pretreatment to CNF/poly(ethylene glycol)-based hydrogels and showed that the rupture strength and elasticity of the composite hydrogel were improved significantly because of the hydrogen bonding and physical entanglement between the CNF and the copolymer.

Polyvinylpyrrolidone (PVP) is a synthetic copolymer widely used in pharmaceutical and biomedical industries for drug-delivery, wound dressing hydrogel [16,17], food packaging [18], and wearable sensors [19]. Despite the fact that PVP hydrogels have beneficial properties including high transparency, biocompatibility, and non-toxic properties, they suffer from poor mechanical performance [20,21]. To improve hydrogel performance for wound dressing, Wang et al. [22] incorporated carboxymethyl cellulose (CMC) or chitin into PVP hydrogel and found that the PVP/CMC blend hydrogel exhibited better gel strength, flexibility, and transparency than pure PVP hydrogel. The addition of carboxymethylated chitin into PVP hydrogel also improved mechanical and swelling properties [23]. The addition of CNF to PVP hydrogel may improve the mechanical strength of PVP hydrogel with small amount of addition. However, CNF has not been studied as an additive of PVP hydrogel. As the properties of CNF are affected by pretreatment methods, it is important to investigate the influence of CNF characteristics on the hydrogel properties in order to select a proper CNF type for PVP hydrogel with improved performance.

In this study, the influence of carboxymethylation and quaternization that introduce anionic and cationic groups on the pulp fibers, respectively, on the properties of CNF were investigated. The absolute charge levels of the chemically pretreated CNF were adjusted by controlling the chemical reaction conditions. The CNF-added PVP hydrogels were prepared by electron beam radiation techniques, and the properties of CNF-added PVP hydrogels, such as gel content, absorptivity of buffer solution and storage modulus, were evaluated to understand the effect of the functional groups of CNF on the properties of wound dressing hydrogels.

## 2. Materials and Methods

### 2.1. Raw Materials

Never-dried bleached eucalyptus kraft pulp provided by Moorim P&P (Ulsan, Korea) was used as a raw material. The chemical composition of the pulp fiber was measured according to a TAPPI method (T203 om-93). The pulp fiber consisted of 79.4 ± 0.6% cellulose, 18.8 ± 0.2% hemicellulose, and trace amounts of lignin and ash. For the carboxymethylation and quaternization, monochloroacetic acid (MCA, Sigma-Aldrich, St. Louis, MO, USA), sodium hydroxide (NaOH, Samchun Chemicals, Seoul, Korea), and 2,3-epoxypropyl trimethylammonium chloride (GMA, Sigma-Aldrich, St. Louis, MO, USA) were used as chemical reagents, and isopropanol (IPA, Duksan Reagent, Ansan, Korea) was used as the solvent medium. Polyvinylpyrrolidone (PVP, average molecular weight 40,000, Sigma-Aldrich, St. Louis, MO, USA) was used to prepare the hydrogel.

### 2.2. Chemical Pretreatment of Pulp

In this study, quaternization was conducted in a water-IPA system, which can prevent the hydrolysis reaction of GMA [24,25]. Firstly, 20 g of the disintegrated pulp fiber suspension was dewatered using a vacuum filtration system to 29 wt%. The pulp fiber was transferred to a polyethylene bag, followed by the addition of 120 mL IPA containing 10 wt% (based on oven dried pulp weight) NaOH. Next, 10 mmol/g GMA was added to the pulp paste. The reaction was carried out for 1 h at 45 °C. In the case of carboxymethylation, the reaction was carried out according to the method described by Im et al. [26]. Then, 20 g of pulp fiber was added to 2 L IPA containing 4 mmol/g of NaOH for alkalization. Thereafter, 1 mmol/g MCA was added to this reaction chamber for esterification, which was carried out at 65 °C for 60 min. After chemical pretreatment, the pretreated pulp was washed with deionized water until the pH and conductivity reached 7 ± 0.5 and ≤20 µS/cm, respectively.

### 2.3. Preparation and Characterization of CNF

A grinder (Super Masscolloider, Masuko Sangyo Co., Ltd., Kawaguchi, Japan) was used to prepare the CNF suspension. The consistency of the untreated and chemically pretreated pulps was adjusted to 0.5 wt% for CNF production. The operation speed and the gap distance of the grinder were adjusted to 1500 rpm and −80 µm, respectively. Untreated CNF (U-CNF), carboxymethylated CNF (CM-CNF), and quaternized CNF (Q-CNF) were prepared by grinding the untreated pulp, carboxymethylated pulp, and quaternized pulp, respectively. The number of the passes through the grinder were presented in Table 1. When the pulp fiber was carboxymethylated or quaternized, seven passes were sufficient to complete nano-fibrillation, while the untreated pulp required 30 passes through the grinder, which indicated that the fibrillation energy was strongly influenced by the charged groups introduced by chemical pretreatments irrespective of the charge type [26].

The carboxyl group content of the carboxymethylated pulp was calculated using a conductometric titration method in accordance with SCAN-CM 65:02. Briefly, carboxymethylated pulp was converted from the sodium form to the proton form using 0.001 mol/L hydrochloric acid, and then titrating with 0.05 mol/L NaOH. The carboxyl group content was calculated based on the oven-dried weight of pulp. The cationic group of the quaternized CNF was evaluated by conductometric titration of chloride ions according to the method described by Pei et al. [27]. The 0.05 g of quaternized CNF was dispersed in 100 mL deionized water under gentle stirring. Then, it was titrated with 5 mM silver nitrate (AgNO_3_, Sigma-Aldrich, St. Louis, MO, USA) solution. The conductivity was measured with adding AgNO_3_ solution, and the quaternary ammonium group content was calculated from the volume of AgNO_3_ titrant added.

The zeta-potentials of the CNF suspensions (0.1%) were evaluated using a Zetasizer Nano ZS (Malvern Panalytical Ltd., Malvern, UK).

The morphological properties of three types of CNF (U-CNF, CM-CNF, and Q-CNF) were investigated using a field emission scanning electron microscope (FE-SEM, Carl Zeiss, Oberkochen, Germany). To prepare the CNF specimen for FE-SEM, a highly diluted suspension (0.003 wt%) was deposited on the glow-discharged carbon grid. Then, the specimen was sputtered with platinum for 100 s at 20 mA using a sputter coater (BAL-TEC SCD 005, Capovani Brothers Inc., Los Angeles, CA, USA) and observed. The width of the CNFs was measured using Image-J software (Version 1.47, University of Wisconsin, Madison, WI, USA). The average width of fibrils and width distribution were obtained from the measurements of at least 100 fibrils.

The transmittances of the CNF suspensions (0.1%) were measured at the wavelength range of 400 to 800 nm using a UV-Vis spectrophotometer (Cary 100, Agilent, Santa Clara, CA, USA).

The crystallinity of CNF was also determined. The measurement of crystallinity of CNF was carried out using an X-ray diffractometer (XRD, Bruker, Karlsruhe, Germany) with a Cu Kα X-ray source set at 5–40° with a scanning speed of 0.5 s/step. The crystallinity was calculated according to Equation (1).

Crystallinity (%) = ((I_200_ − I_AM_))/I_200_) × 100 (1)

where I_200_ is the intensity of the 200 peak at 2θ = 22.7°, and I_AM_ is the minimum intensity between the 200 and 110 peaks at 2θ = 18°.

The rheological properties of the CNF suspension were evaluated using a Bohlin rheometer (CVO, Malvern Instruments Ltd., Malvern, UK) with a gap angle between the cone and plate of 4° and a cone diameter of 4 cm. The rotational viscosity with an increase in shear rate was measured using 0.5% CNF suspensions. An oscillatory rheology was evaluated to understand the network properties of CNF suspensions. An amplitude sweeps were carried out to evaluate the rheological properties of CNF. The shear-stress-amplitude sweep was conducted in the range of 0.5–100 Pa at a constant frequency of 1 Hz.

### 2.4. Preparation of CNF-Added PVP Hydrogels

PVP hydrogels containing different types of CNFs were prepared using electron beam radiation technique. This technique does not require an additional purification process to remove the crosslinking agent used for typical manufacture of hydrogel. When PVP in an aqueous solution is irradiated, most of the energy is absorbed by the water. After that, hydrated electrons, hydroxyl radicals, and hydrogen atoms are formed by the ionized water. Among these, hydroxyl radicals strongly react with the PVP polymer generating macro-radicals which result in the crosslinking of PVP polymers [28]. First, the CNF suspensions were diluted using deionized water from 0.01 to 0.1 wt%. Next, PVP (5 wt%) was added to the CNF suspension, and the mixture was stirred for 1 h at 50 °C. Then, 100 g of the mixed solution was poured into a petri dish. Electron-beam irradiation was performed with a 10 MeV linear electron accelerator (MB 10-8/635). Irradiation was carried out using 8 kW beam power and a 20 kGy exposure dose was applied to the samples. As a control, PVP hydrogel without CNF was prepared in the same manner. The structure of PVP hydrogel and CNF-added PVP hydrogels was observed using FE-SEM. To prepare the specimens without structure collapse, hydrogels were frozen using liquid nitrogen and freeze-dried at −78 °C and 5 mTorr. The cross-section of the dried hydrogel was coated with platinum using a sputter coater and then observed using FE-SEM.

### 2.5. Characterization of CNF-Added PVP Hydrogels

The extent of cross-linking in hydrogels was evaluated from the gel content. Circular gel disc samples with 8 mm in diameter were prepared using a biopsy punch. The hydrogel samples were dried in a vacuum oven at 70 °C to a constant weight (W_i_). The dried samples were then immersed in deionized water at room temperature (25 °C) for 24 h for sol extraction. The undissolved parts were dried again at 70 °C to a constant weight (W_d_). The gel content was calculated by Equation (2).

Gel content (%) = (W_d_/W_i_) × 100 (2)

where W_i_ and W_d_ are the initial weight (g) of the dried gel and the weight (g) of the dried gel after extraction, respectively.

Hydrogel absorptivity was determined using a phosphate buffered saline (PBS) solution. The dried gels were immersed in a pH 7.4 PBS solution for 24 h. The swollen weight (g) of the hydrogel (W_s_) was measured, and the absorptivity was evaluated according to Equation (3).

Absorptivity (%) = ((W_s_ − W_i_)/W_i_) × 100 (3)

To evaluate the storage modulus of hydrogels before and after immersion for 16 h in the PBS solution, a digital rheometer (HAAKE MARS III, Thermo Scientific, Karlsruhe, Germany) was used. The gel modulus was measured using a parallel plate geometry (8 mm) with a nominal force of 0.2–0.3 N and gap size of 0.3 mm. The average gel storage modulus of each sample was calculated from the linear viscoelastic region.

## 3. Results

### 3.1. Characteristics of CNF Prepared under Different Conditions

**Figure 1:**
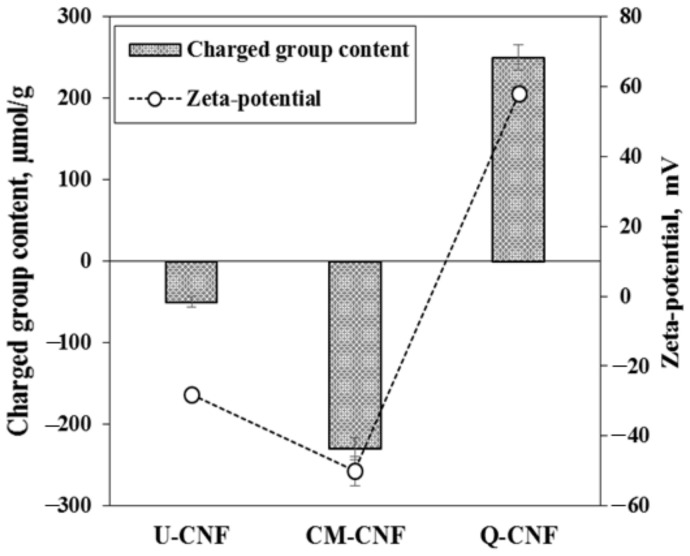
Charge properties of CNF prepared under different pretreatment conditions.

The characteristics of three types of CNFs (U-CNF, CM-CNF, and Q-CNF) were evaluated. As shown in Figure 1, the charged group content and zeta-potential, indicating the electrostatic characteristics of the cellulose surface, were changed by chemical pretreatment. The untreated pulp had a carboxyl group content of −50 µmol/g, which originated from hemicelluloses and cellulose of the bleached kraft pulp. Bleached kraft hardwood pulp has carboxyl groups such as hexenuronic acid generated from pulping and bleaching processes [29]. The zeta-potential of CM-CNF exhibited approximately −40 mV, whereas the Q-CNF had approximately +70 mV of zeta-potential. To examine the effect of the ionicity of chemically pretreated CNF on the PVP hydrogel, the charged group content was adjusted to 240 µmol/g by controlling the reaction condition in each pretreatment (Figure 1).

**Figure 2:**
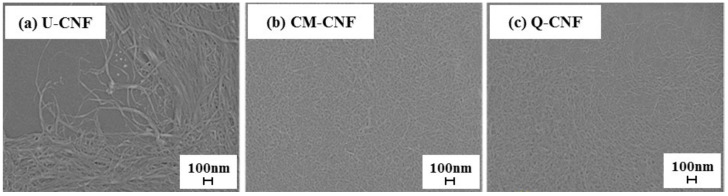
SEM images of (**a**) untreated cellulose nanofibrils (U-CNF), (**b**) carboxymethylated cellulose nanofibrils (CM-CNF), and (**c**) quaternized cellulose nanofibrils (Q-CNF).

**Figure 3:**
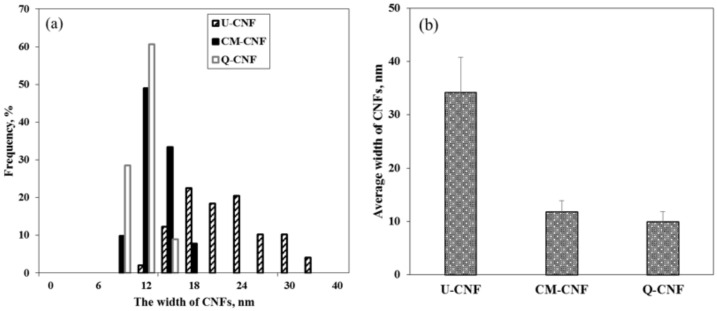
Morphological properties of U-CNF, CM-CNF, and Q-CNF: (**a**) width distribution curve and (**b**) average width.

Figure 2 and Figure 3 show the morphology, width distribution and average width of CNFs prepared under different treatment conditions. In the case of U-CNF, large-sized fibril bundles still existed after 30 grinding passes, which can be clearly seen in the SEM image. Therefore, U-CNF had the largest average width as well as a wide width distribution of fibrils, ranging from 11.6 nm to 35.2 nm. On the other hand, the chemically pretreated CNFs, including CM-CNF and Q-CNF, were completely nanofibrilized without any fibril bundles. In particular, Q-CNF had the most narrow and uniform width about 9 nm. This result indicates that high surface charges of the fiber promoted the production of thinner and more uniform fibrils [30].

**Figure 4:**
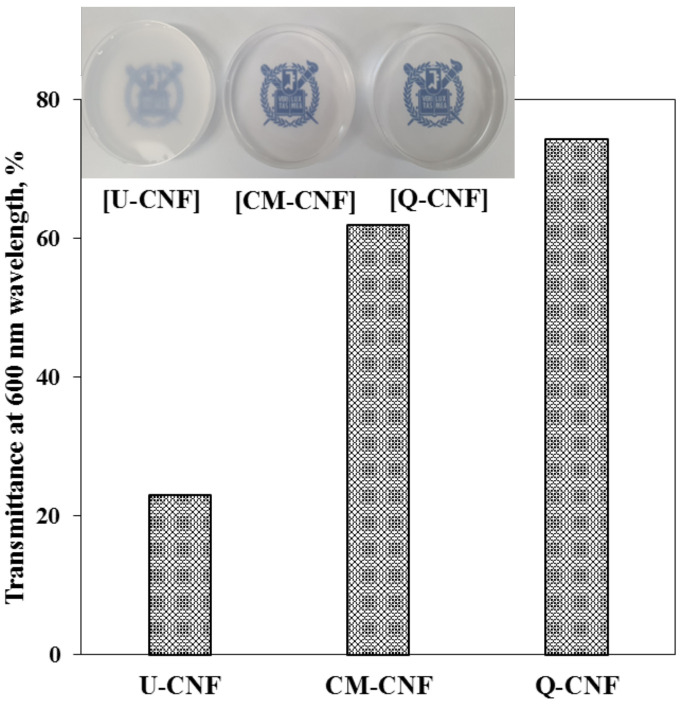
Appearance and transmittance of 0.1% CNF suspensions.

In addition, the morphological differences of fibrils affected the appearance and an optical transparency of CNF suspensions at a wavelength of 600 nm as shown in Figure 4. The highest transmittance was achieved with Q-CNF because of its thin and more uniform width as shown in Figure 3.

**Figure 5:**
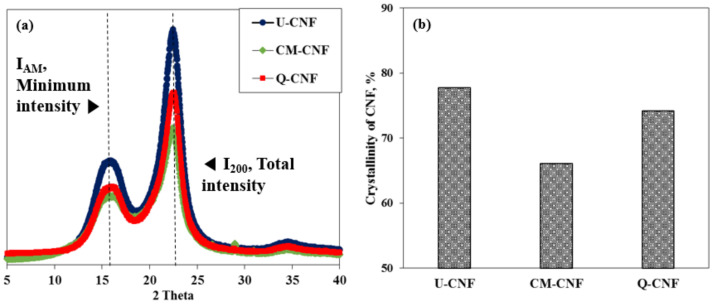
(**a**) XRD pattern and (**b**) crystallinity of U-CNF, CM-CNF, and Q-CNF.

Figure 5 shows the results of XRD analysis. All CNF types exhibited typical diffraction peak profile of cellulose I [31]. Although the similar amounts of opposite charged group were introduced into the cellulose by the chemical pretreatments, the carboxymethylation pretreatment had a greater influence on the crystallinity of cellulose than quaternization pretreatment. In the quaternization process, the addition of sodium hydroxide not only accelerates the reactivity between the GMA and cellulose but also suppresses side reactions with GMA [32]. However, in the case of carboxymethylation, the sodium hydroxide reacts with hydroxyl groups of cellulose through mercerization, and the alkali-swollen cellulose then becomes accessible and reactive towards the carboxymethylation agent [33], which leads to reduction in crystallinity of CNF.

**Figure 6:**
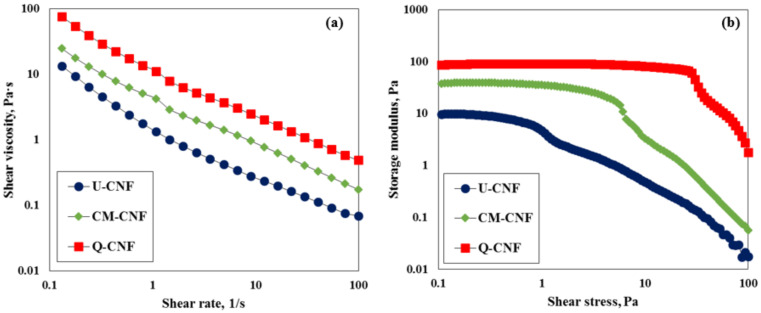
Rheological properties of CNF suspensions: (**a**) shear viscosity and (**b**) storage modulus as a function of stress.

Figure 6 shows the rheological properties of each CNF suspension (0.5 wt%). All CNF suspensions showed shear thinning behavior (Figure 6a), because the interactions between fibrils orient the fibrils along the direction of shear [34]. The measured shear viscosity increased in the order of Q-CNF > CM-CNF > U-CNF. Amplitude sweep mode measurements were also carried out to find the network strength of CNF suspension. The storage modulus of three CNF suspensions showed a plateau region in a low level range of shear stress. However, the storage modulus of CNF suspensions decreased over a certain stress level, indicating the breakage of CNF network and liquid-like flow behavior. The yield stress could be determined by the intercept between two tangential lines of storage modulus. The Q-CNF suspension had a higher yield stress than CM-CNF and U-CNF (Q-CNF: 60.9 Pa; CM-CNF: 16.4 Pa; U-CNF: 4.2 Pa), most probably because the Q-CNF had the narrowest average width. The aspect ratio of CNF is likely to be another reason for the increase in yield stress [35,36,37].

### 3.2. Characteristics of CNF-Added PVP Hydrogel

**Figure 7:**
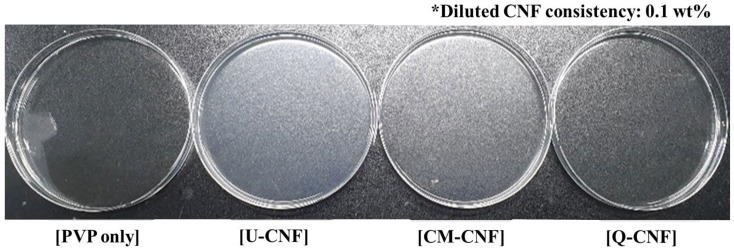
Appearance of pure polyvinylpyrrolidone (PVP) hydrogel and CNF-added PVP hydrogels.

**Figure 8:**
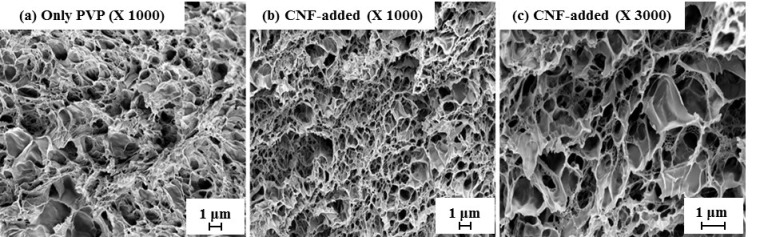
Cross-section of (**a**) pure PVP hydrogel and (**b**,**c**) U-CNF-added PVP hydrogel.

Use of CMC [21] and carboxymethylated chitosan [23] in PVP/polysaccharide blended hydrogels have been investigated and shown that CMC or chitosan can be physically cross-linked with PVP by irradiation treatments. However, the use of CNF for PVP hydrogel has not been made yet. In this study, PVP was added into the CNF suspensions of which consistencies were controlled from 0.01% to 0.1%. Then, CNF-added PVP hydrogel was prepared by electron beam irradiation to use it for wound dressing. The properties of the hydrogels including gel strength, swelling behavior, and mechanical property were investigated. Figure 7 showed the appearance of the pure PVP hydrogel and CNF-added PVP hydrogels. The U-CNF-added PVP hydrogel exhibited the lowest transparency compared with other hydrogels, which means that the transparency of hydrogel was influenced by the transmittance of CNF as shown in Figure 4. Table 2 shows the gel content of CNF-added PVP hydrogels. The gel contents of hydrogels were about 85% for the whole range of CNF consistencies, irrespective of the type of CNF and addition level. Generally, the addition of polysaccharides decreases the gel content of a hydrogel because the polysaccharide acts as a crosslinking inhibitor whereby the PVP competes with the polysaccharide for the free radicals [23,38]. However, the gel contents of the CNF-added hydrogels were maintained at approximately 85% because a very small amount of CNF was contained in the hydrogels. Cross-sections of the pure PVP hydrogel and the CNF-added hydrogels were examined using FE-SEM and shown in Figure 8. To preserve the structure of the hydrogels, they were dried using a freeze-drying process. It shows that all hydrogels exhibited a random three-dimensional network regardless of CNF addition. CM-CNF-added PVP hydrogel and Q-CNF-added PVP hydrogel had similar network structure to U-CNF-added PVP hydrogel.

**Figure 9:**
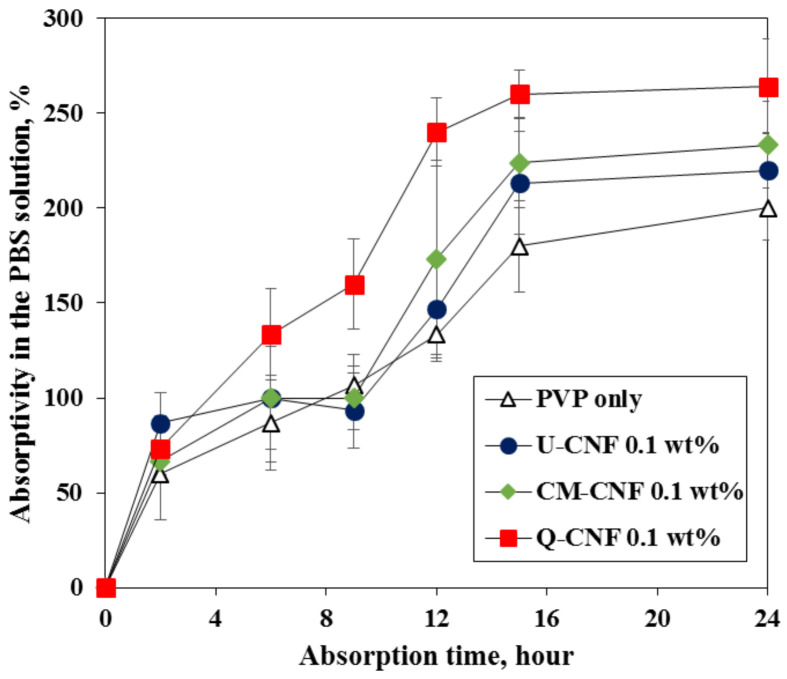
Absorptivity of hydrogels in phosphate buffered saline (PBS) solution for 24 h.

The absorptivity of hydrogels was evaluated by impregnating them in the PBS solution for 24 h. The PBS solution is the most commonly used swelling medium to simulate hydrogel behavior in wound dressing [39], because the ions in PBS can move freely into the hydrogels. Thus, it quickly reduces the difference in the osmotic pressures between the hydrogel and the surrounding solution. Zhao et al. [23] showed that the swelling degree of the hydrogel increases when CMC is added to the PVP network due to the high hydrophilicity of polysaccharides. The addition of CNF also showed the same tendency as shown in Figure 9. The addition of three types of CNFs increased the absorptivity of hydrogel in the PBS solutions, and the maximum absorptivity was achieved after 15 h irrespective of the type of CNF. High absorptivity of the hydrogel in the PBS solution indicates the high absorptivity of exudates, which is greatly effective to keep the wound site dry and prevent air-borne infection [40]. The Q-CNF-added PVP hydrogel showed the highest absorptivity than the other CNF-added hydrogels. In particular, the absorptivity of the Q-CNF-added hydrogel was 48% higher than the PVP hydrogel after 15 h. It is worth noting that there was no significant difference in the average width between CM-CNF and Q-CNF. Therefore, high absorptivity of Q-CNF-added PVP hydrogels can be explained by the electrostatic attraction between cationic groups on Q-CNF fibrils and the polyvalent phosphate anions of PBS solution rather than morphological effect. High water holding capacity of Q-CNF may also contribute to increase the swelling degree. In addition, a large specific surface area of CM-CNF appeared to contribute to increasing the absorptivity of CM-CNF-added PVP hydrogel compared to U-CNF-added PVP hydrogel.

**Figure 10:**
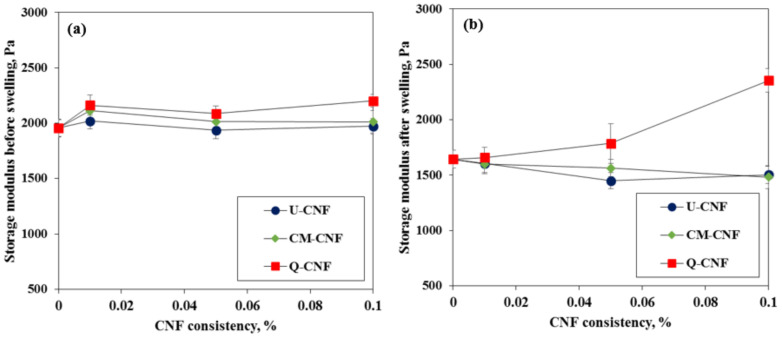
Average storage modulus: (**a**) before immersion and (**b**) after immersion in PBS solution after 16 h.

The storage modulus of hydrogels was evaluated. Figure 10a shows the storage modulus prior to immersion in the PBS solution depending on the type of CNF and the consistency of CNF suspension. The addition of CNF did not have a significant effect on the average storage modulus of the hydrogels before swelling, which were around 2000 Pa. The Q-CNF-added PVP hydrogel had a little higher storage modulus. However, the storage modulus of pure PVP hydrogel decreased from approximately 1900 Pa to 1650 Pa after immersion in the PBS solution (Figure 10b). Slight decreases of the storage modulus of the blended PVP hydrogels were obtained as the addition amount of U-CNF and CM-CNF increased. On the other hand, the addition of Q-CNF exhibited totally different trend from other CNF-added hydrogels after immersion as shown in Figure 10b. Sim et al. [41] studied the network strength of anionic CNF suspensions depending on the presence of salts with bivalent cations. They found that the storage modulus and yield stress of anionic CNF suspensions increased with the addition of a salt solution due to fibril coagulation caused by charge neutralization. Therefore, an increase in storage modulus of hydrogel with an increase of Q-CNF content can be attributed to the electrostatic interaction between cationic groups of CNF and phosphate ions of the PBS solution. From the storage modulus, the average mesh size which means the distance between valid crosslinking points could be calculated based on the rubber elasticity theory as described in the literatures [42,43]. After immersion in the PBS solution, the average mesh sizes were 13.6 nm and 12.0 nm for the pure PVP hydrogel and Q-CNF (0.1% consistency)-added PVP hydrogel, respectively. The small mesh size of hydrogel indicates high crosslinking density. Therefore, it indicates that Q-CNF increased the crosslinking density of the hydrogel by electrostatic interaction.

## 4. Conclusions

The influence of CNF characteristics including morphological and electrostatic properties on the properties of CNF-added PVP hydrogels were investigated. Untreated CNF, carboxymethylated CNF, and quaternized CNF were prepared by different chemical pretreatment and grinding process. The passage number of grinding required to produce CNF substantially decreased by introduction of charged group to pulp fibers. Chemical pretreatments gave CNF having more narrow and uniform width. These morphological differences affected the appearance and optical transparency of CNF suspension. The highest optical transparency was achieved with Q-CNF. In addition, the crystallinity of Q-CNF and the viscosity and storage modulus of Q-CNF suspension were significantly higher than carboxymethylated CNF. Using these different types of CNFs, transparent CNF-added PVP hydrogels were prepared by electron beam irradiation treatment. The gel content and internal structure of CNF-added PVP hydrogel were similar irrespective of the type of CNF and their addition level. However, the absorptivity and storage modulus after swelling in the PBS solution were significantly affected by the electrostatic properties of CNF. Q-CNF-added PVP hydrogel exhibited the highest absorptivity and the improved storage modulus even after immersion in PBS solution due to electrostatic interaction between electrolytes and cationic groups on the quaternized CNF. These results indicated that the electrostatic characteristics of CNF is important as much as the morphological properties of CNF to improve the properties of hydrogel when incorporating CNF into PVP hydrogel.

## Figures and Tables

**Table 1:** Classification of cellulose nanofibrils (CNF) depending on the pretreatment type and number of grinding passes required to complete fibrillation.

Classification	Pretreatment	Number of Grinding Passes
U-CNF	Untreated	30
CM-CNF	Carboxymethylation	7
Q-CNF	Quaternization	7

**Table 2:** Gel content of CNF-added PVP hydrogels depending on the type of CNF and suspension consistency.

Classification	Gel Content (%) of Pure PVP Hydrogel (without CNF)	Gel Content (%) of CNF-Added PVP Hydrogel (CNF Consistency)		
		0.01%	0.05%	0.1%
U-CNF	85.4	84.7	86.2	85.1
CM-CNF		86.8	84.4	84.7
Q-CNF		85.7	82.5	84.8

## Data Availability

The data presented in this study are available on request from the corresponding author. The data are not publicly available due to the author’s readiness to provide it on request.
